# Short- and long-term renal outcomes following severe rhabdomyolysis: a French multicenter retrospective study of 387 patients

**DOI:** 10.1186/s13613-020-0645-1

**Published:** 2020-03-02

**Authors:** Nelly Candela, Stein Silva, Bernard Georges, Claire Cartery, Thomas Robert, Julie Moussi-Frances, Eric Rondeau, Jean-Michel Rebibou, Laurence Lavayssiere, Julie Belliere, Thierry Krummel, Céline Lebas, Olivier Cointault, Marion Sallee, Stanislas Faguer, on behalf of the French Intensive Care Renal Network (F.I.R.N)

**Affiliations:** 1 Département de Néphrologie et Transplantation d’organes, Hôpital Rangueil, Intensive Care Unit, Centre Hospitalo-Universitaire de Toulouse, 1, Avenue Jean Poulhes, 31059 Toulouse, France; 2 Réanimation–URM, Hôpital Purpan, Centre Hospitalo-Universitaire de Toulouse, Toulouse, France; 3 Département d’Anesthésie et Réanimation–Unité de Réanimation Polyvalente, Hôpital Rangueil, Centre Hospitalo-Universitaire de Toulouse, Toulouse, France; 4 Service de Néphrologie, Centre Hospitalier de Valenciennes, Valenciennes, France; 5 Service de Néphrologie et Transplantation Rénale, Hôpital de la Conception, Centre Hospitalo-Universitaire de Marseille, Marseille, France; 6 Service d’Urgences Néphrologiques et Transplantation Rénale, Hôpital Tenon, Assistance Publique des Hôpitaux de Paris, Paris, France; 7 Service de Néphrologie, Centre Hospitalo-Universitaire de Dijon, Dijon, France; 8 Service de Néphrologie, Centre Hospitalo-Universitaire de Strasbourg, Strasbourg, France; 9 Service de Néphrologie, Centre Hospitalo-Universitaire de Lille, Lille, France; 10 Institut National de la Science et de la Recherche Médicale, Institut National de la recherche Agronomique, Université Aix-Marseille, C2VN, Marseille, France; 11 Institut National de la Science et de la Recherche Médicale, Unité 1048 (équipe 12–Fibrose rénale: détection et mécanismes de progression), Paris, France

**Keywords:** Rhabdomyolysis, Acute kidney injury, CKD progression, Outcomes, Myoglobin

## Abstract

**Background:**

Rhabdomyolysis is a life-threatening disease that can lead to severe hyperkalemia, acute kidney injury (AKI) and hypovolemic shock. The predictive factors of AKI and acute to chronic kidney disease (CKD) transition remain poorly described.

**Methods:**

This multicenter retrospective study enrolled 387 patients with severe rhabdomyolysis (CPK > 5000 U/L). Primary end-point was the development of severe AKI, defined as stage 2 or 3 of KDIGO classification. Secondary end-points included the incidence of AKI to CKD transition.

**Results:**

Among the 387 patients, 315 (81.4%) developed AKI, including 171 (44.1%) with stage 3 AKI and 103 (26.6%) requiring RRT. Stage 2–3 AKI was strongly correlated with serum phosphate, potassium and bicarbonate at admission, as well as myoglobin over 8000 U/L and the need for mechanical ventilation. 42 patients (10.8%) died before day 28. In the 80 patients with available eGFR values both before and 3 months after the rhabdomyolysis, the decrease in eGFR (greater than 20 mL/min/1.73 m^2^ in 23 patients; 28.8%) was correlated to the severity of the AKI and serum myoglobin levels > 8000 U/L at admission.

**Conclusions:**

Severe rhabdomyolysis leads to AKI in most patients admitted to an ICU. Mechanical ventilation and severity of the rhabdomyolysis, including myoglobin level, are associated with the risk of stage 2–3 AKI. The long-term renal decline is correlated to serum myoglobin at admission.

## Background

Rhabdomyolysis is a muscular disorder characterized by the leakage of skeletal muscle-cell contents (electrolytes, myoglobin and sarcoplasmic proteins) into circulation. Trauma (crush syndrome) is the leading cause of rhabdomyolysis, followed by medical or surgical conditions (heat stroke, immobilization, major artery occlusion, infections, status epilepticus, drugs or genetic defects) [1–3]. Beside the severity of the acute condition that led to rhabdomyolysis, life-threatening complications related to the massive muscle cells necrosis include severe hyperkalemia and hypocalcemia, acute kidney injury and hypovolemic shock [4]. It is worth noting that in the most severe forms of rhabdomyolysis, mortality rates may reach up to 59% of patients [5].

Acute kidney injury (AKI) occurs in 19–58% of patients with rhabdomyolysis, depending on the diagnostic criteria of AKI and the severity of the rhabdomyolysis [6–8]. Mechanisms underlying rhabdomyolysis-related AKI are complex and intertwined. Experimental evidence suggests that free myoglobin induces intrarenal vasoconstriction (activation of the vasopressin, renin–angiotensin and sympathetic nervous systems; nitric oxide defect), direct tubule injury, tubular obstruction and macrophage-dependent inflammation [4, 7, 9, 10]. Whether cell lysis-related hyperphosphoremia and subsequent calcium–phosphorus deposition in the kidney may also promote AKI is currently unknown. In addition, fluid sequestration within the damaged muscle mass is accompanied by intravascular volume depletion and subsequent worsening of the AKI. Ultimately, 8–65% of patients with rhabdomyolysis will require renal replacement therapy (RRT) [3–5]. After the episode, renal recovery is observed in most survivors but data on the risk of AKI to chronic kidney disease (CKD) transition in this specific setting are scarce [4, 11].

Until recently, the risk of AKI or death was primarily estimated using the maximum peak of serum creatine phosphokinase (CPK), with a suggested discriminative cut-off of 5000 U/L [12, 13]. However, recent retrospective studies have stressed the low predictive value of the maximum peak of CPK as isolate predictive biomarker and alternatively proposed to integrate this parameter within a composite model [3, 5]. Nevertheless, it should be highlighted that, due to its registry-based design, this study did not take into account important factors related to patients condition (e.g., incidence of AKI independent of the need for RRT or treatment) or ICU-related condition (i.e., use of mechanical ventilation or vasoactive agents) [14, 15].

This large, multicenter study, including 387 patients from eight intensive care units in France, aimed to characterize the incidence of AKI in patients with severe rhabdomyolysis, to identify clinically useful predictive factors of KDIGO stage 2–3 AKI, and wanted to provide a special focus over the risk of AKI to CKD transition in these challenging setting.

## Patients and methods

This study retrospectively included adult patients referred, between January 2004 and July 2017, for severe rhabdomyolysis to eight intensive care units, located in six University teaching Hospitals in France. To be included, minimum serum CPK levels had to be higher than 5000 U/L in 72 h following admission. Patients were identified using the computerized medical system in each unit. Patients with pre-existing end-stage renal disease were excluded from the analysis. According to the French law on retrospective observational studies, and the recommendations of our Institutional Review Board, the written informed consent requirement was waived (IRB approval reference no OSB/MEL/IO/2019-1518).

### Objectives and outcomes

The main objective of the study was to describe the incidence of AKI after severe rhabdomyolysis. Secondary objectives included the identification of predictive factors of KDIGO stage 2–3 AKI, the description of AKI to CKD transition and the role of serum myoglobin concentration as a risk factor of transition toward CKD.

### Data collection and definitions

Habitus, drug exposure and organ supportive care were reviewed. The causes of the rhabdomyolysis were categorized as crush, prolonged immobilization, ischemic disease, status epilepticus, malignant hyperthermia and other. The following laboratory values were collected at admission: creatinine, myoglobin, CPK, potassium, calcium, phosphate, bicarbonates, lactates, white blood cell count, hemoglobin, platelets and prothrombin time. AKI was defined according to the Kidney Disease: Improving Global Outcomes (KDIGO) group, using creatinine values [16]. Accordingly, AKI was defined by the increase of serum creatinine in the 7 days following the onset of rhabdomyolysis. Patients with overt stage 2 or 3 AKI at the admission were excluded from the statistical analyses. Due to the retrospective design of the study, RRT was started according to the local unstandardized practice. The peaks of serum creatinine and CPK were also collected. Organ support (mechanical ventilation and vasoactive drugs) were considered at day 1. Chronic kidney disease was staged according to the KDIGO classification [17] and CKD progression was estimated by measuring the decrease of eGFR at month 3 (i.e., month 3 eGFR minus basal eGFR). To assess accurately the CKD progression, we only studied the group of patients with eGFR available before rhabdomyolysis (basal eGFR) and at month 3 (± 2 weeks).

### Statistical analysis

Continuous variables were given as median and interquartile range, while categorical variables were given as number and percentage. Univariate (unadjusted) analysis of KDIGO stage 2–3 AKI was performed using the Mann–Whitney *U* test for continuous variables and the Fischer exact test for categorical variables. For stage 2–3 AKI prediction, variables with a *p* value below 0.1 by univariate analysis were included in the multivariate analyses and exited from the model if *p* value was > 0.2. Two models including prothrombin time and serum phosphate, calcium, myoglobin, creatine phosphokinase, potassium or bicarbonate at the admission, as well as the need of vasoactive drugs or mechanical ventilation were studied. Adjusted odds hazard ratios were estimated by multivariable (step-by-step descending) logistic regression. To be considered significant, the *p* value had to be lower than 0.05. Missing values were excluded from the analysis. Statistical analyses were performed using Xlstat software.

## Results

### Patients’ characteristics

**Fig. 1:**
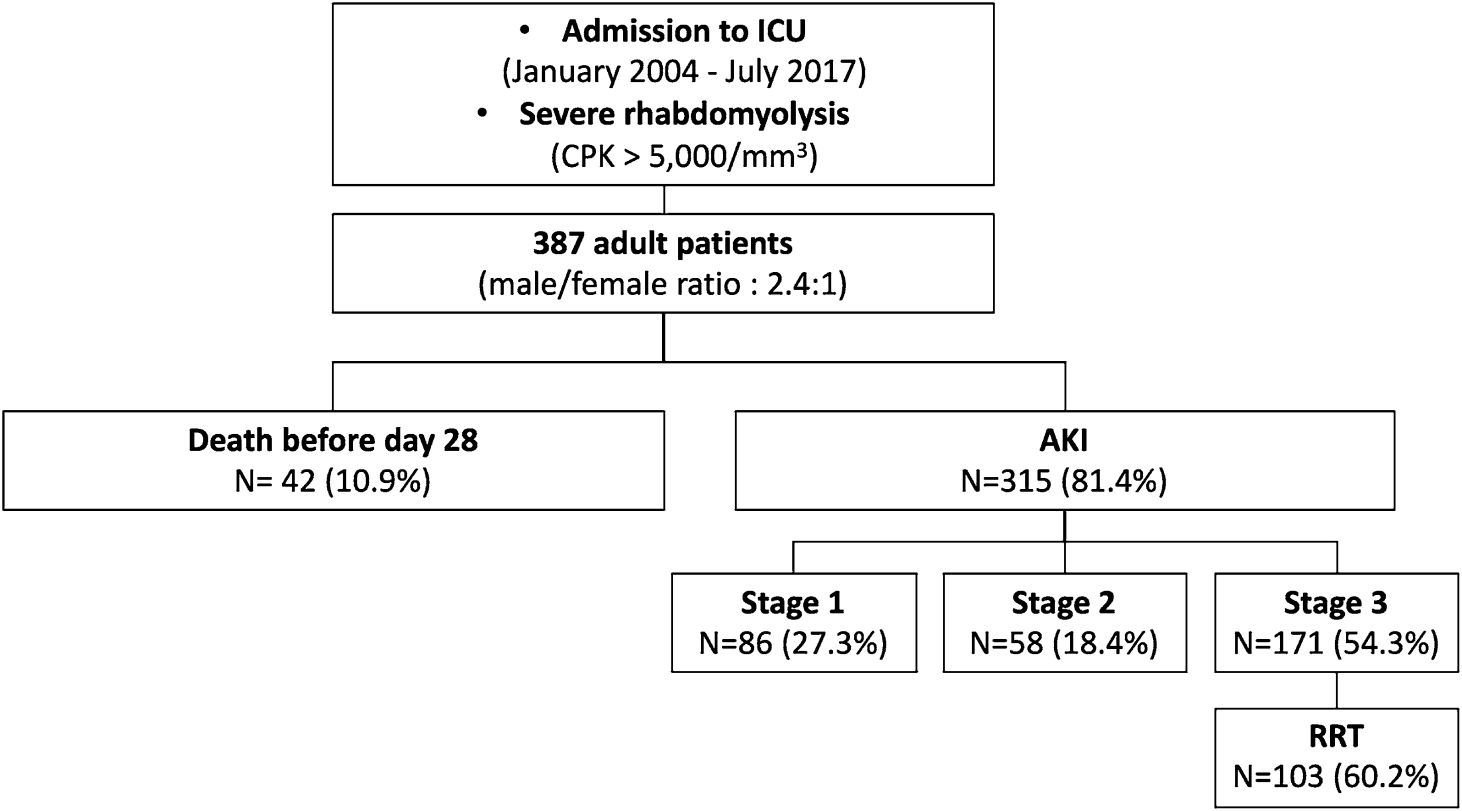
Flowchart of the study. *RRT* renal replacement therapy

Three hundred and eighty-seven patients were included in the study (median age 49 years, IQR [34; 62]; female gender *n* = 115 (29.7%)) (Table 1 and Fig. 1). The estimated glomerular filtration rate (eGFR) before admission was available for 186 individuals. Median baseline eGFR was 92 mL/min/1.73 m^2^ (IQR [71; 110]), and eGFR was higher than 60 mL/min/1.73 m^2^ in 159 individuals (85.5%; median serum creatinine 76 μmol/L, IQR [62; 91]).

**Table 1:** Characteristics of 387 patients with severe rhabdomyolysis

	Population, *n* = 387	Acute kidney injury stage 2–3		*p* value
		Yes, *n* = 229 (%)	No, *n* = 158 (73.4%)	
Age (years)	48.8 (34.5–62.5)	50 ± 18	47 ± 21	0.09
Female gender (*n*, %)	115 (29.7)	68 (29.7)	47 (29.7)	1.00
Past medical history				
Drugs abuse (*n*, %)	75 (19.3)	21 (9.2)	9 (5.7)	0.25
Diabetes mellitus (*n*, %)	30 (7.7)	29 (12.7)	10 (6.3)	0.06
Cirrhosis (*n*, %)	27 (6.9)	4 (1.7)	1 (0.6)	0.65
RAAS blocking agents (*n*, %)	5 (1.2)	44 (19.2)	20 (12.6)	0.10
Cortisone (*n*, %)	64 (16.5)	8 (3.5)	1 (0.6)	0.09
Diuretics (*n*, %)	9 (2.3)	20 (8.7)	7 (4.4)	0.14
Characteristics at the admission				
Creatine phosphokinase (×10^3^; IU/L)	8167 [3173–19,604]	31.7 ± 99.7	10.9 ± 15.4	0.0002
Myoglobin (×10^3^; ng/mL)	8433 [3272–21,715]	24.6 ± 41.3	10.1 ± 18.9	< 0.0001
Serum phosphorus (mmol/L)	1.27 [0.89–1.69]	1.58 ± 0.8	1.1 ± 0.52	< 0.0001
Serum calcium (mmol/L)	2.07 [1.83–2.22]	2.01 ± 0.3	2.08 ± 0.3	0.02
Serum potassium (mmol/L)	4.4 [3.9–5.3]	4.9 ± 1.2	4.2 ± 0.8	< 0.0001
Serum bicarbonate (mmol/L)	21 [17–24]	19.3 ± 5.4	2.2 ± 4.8	< 0.0001
Arterial lactates (mmol/L)	2.9 [1.7–4.9]	4.5 ± 3.9	3.3 ± 3.2	0.001
Bilirubin (mmol/L)	10 [6.4–16.3]	75 ± 20	65 ± 22	0.51
Prothrombin time (%)	87 (22.4)	201 ± 91	205 ± 90	< 0.0001
Platelet count (G/L)	194 [137–255]	12.6 ± 3.1	14.6 ± 15	0.39
Hemoglobin (g/dL)	12.9 [11–14.7]			0.24
Causes of rhabdomyolysis				0.03
Crush syndrome (*n*, %)	108 (27.9)	52 (22.8)	56 (35.4)	
Vascular ischemia (*n*, %)	71 (18.3)	42 (18.3)	18 (11.4)	
Status epilepticus (*n*, %)	15 (3.9)	7 (3)	8 (5)	
Immobilization (*n*, %)	131 (33.9)	83 (36.2)	48 (30.3)	
Malignant hyperthermia (*n*, %)	7 (1.8)	6 (2.7)	1 (0.7)	
Other (*n*, %)	66 (17)	39 (17)	27 (17.2)	
Life-sustaining treatments at day 1				
Vasoactive drugs (*n*, %)	185 (47.8)	133 (58)	52 (32.9)	< 0.0001
Mechanical ventilation (*n*, %)	229 (59.1)	155 (67.7)	74 (46.8)	< 0.0001

*RAAS* renin–angiotensin–aldosterone system

The main causes of rhabdomyolysis were prolonged immobilization (*n* = 131, 33.8%), crush syndrome (*n* = 108, 27.9%), vascular ischemia (*n* = 71, 18.3%), status epilepticus (*n* = 15, 3.9%), and malignant hyperthermia (*n* = 7, 1.8%). A precipitating factor, such as alcoholism or drug abuse, was identified in 75 (19.3%) and 30 (7.7%) individuals, respectively.

Median follow-up was 8.7 months [1.3; 28.5]. The mortality rate at day 28 was 10.9% (42 patients). Median survival was not reached. Mechanical ventilation and vasopressor drugs were required in 229 (59.1%) and 185 (47.8%) patients.

### Incidence of AKI

Overall, 315 patients (81.4%) developed AKI in the ICU, including 58 (15%) and 171 individuals (44.1%) with stage 2 and 3 AKI, respectively, and 103 (26.6%) requiring renal replacement therapy (including continuous renal replacement therapy in 58 individuals and intermittent hemodialysis in 44 (unknown in one)).

### Predictive factors of stage 2–3 AKI

We aimed to identify the predictive factors of stage 2–3 AKI. As shown in Table 1, 229 patients (59%) developed AKI stage 2–3 during hospitalization. Using univariate analysis, a history of diabetes mellitus, CPK and myoglobin levels at admission, serum phosphates, potassium, bicarbonates, arterial lactates, normalized prothrombin time, the cause of the rhabdomyolysis, and the need for vasoactive drugs and mechanical ventilation were all associated with stage 2–3 AKI. In the 289 patients with available data, sodium bicarbonate (*n* = 134) was more frequently used in patients that ultimately required RRT (47/134 vs. 34/155, *p* = 0.013).

Through multivariate analysis, using a descending step-by-step logistic regression, the need for mechanical ventilation, serum phosphate and potassium at admission, as well as myoglobin over 8000 U/L was predictive of stage 2–3 AKI (Table 2).

**Table 2:** Multivariate analysis of the predictive factors of KDIGO stage 2–3 acute kidney injury in patients with severe rhabdomyolysis

	Model 1 [AUC 0.81] [Hosmer–Lemeshow test 0.31]		Model 2 [AUC 0.82] [Hosmer–Lemeshow test 0.51]	
	Hazard ratio [IC_5–95%_]	*p* value	Hazard ratio [IC_5–95%_]	*p* value
Serum phosphorus (per mmol/L)	2.2 [1.6–4.9]	0.0004	2.8 [1.4–5.6]	0.003
Serum calcium (per mmol/L)	2.37 [0.7–8.3]	0.18	–	–
Myoglobin (> 8000 U/L at admission)	–	–	2.03 [1.01–4.1]	0.02
Creatine phosphokinase (per 1000 UI/L)	1.01 [0.99–1.03]	0.09	–	–
Serum potassium (per mmol/L)	1.5 [1.1–2.0]	0.02	1.7 [1.2–2.4]	0.003
Bicarbonates (mmol/L)	0.92 [0.86–0.99]	0.03	0.95 [0.88–1.02]	0.18
Vasoactive drugs	1.9 [0.8–4.3]	0.12	1.8 [0.8–4.1]	0.16
Mechanical ventilation	2.4 [1.05–5.5]	0.04	1.9 [0.8–4.6]	0.12
Prothrombin time (%)	0.99 [0.97–1.01]	0.20	–	–

### Acute to chronic kidney disease transition

**Fig. 2:**
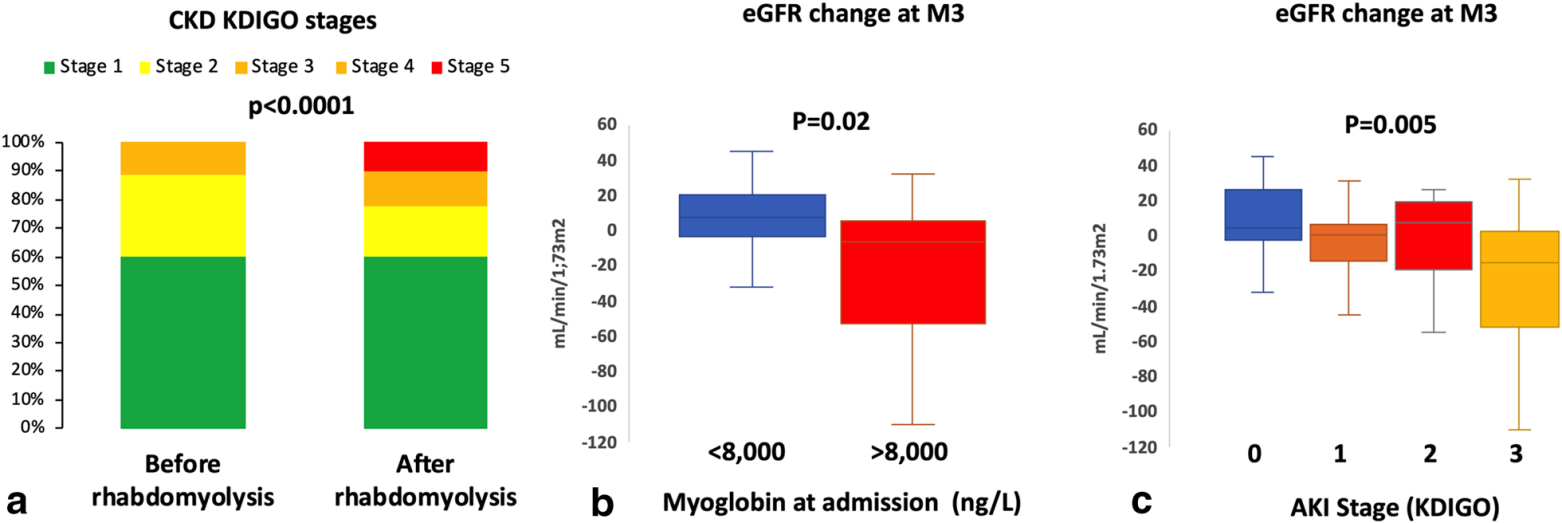
Risk of acute kidney injury to chronic kidney transition after an episode of severe rhabdomyolysis. **a** CKD KDIGO stages before and after (month 3) severe rhabdomyolysis. **b**, **c** Change of the estimated glomerular filtration rate 3 months after a severe rhabdomyolysis (eGFR at month 3 minus basal eGFR) according to the serum level of myoglobin at admission (**b**) and the severity of the acute kidney injury (**c**)

In the 259 patients monitored for longer than 2 months and with available eGFR values before the rhabdomyolysis (median 96 mL/min/1.73 m^2^, IQR [79; 109]), eGFR at month 3 was available for 80 patients (31%; median 91 mL/min/1.73 m^2^, IQR [56; 112]). Among these 80 patients, 12, 18, 12 and 38 developed AKI stage 0, 1, 2 and 3 after their admission to ICU, respectively. The median decrease in eGFR at month 3 was − 2.5 mL/min/1.73 m^2^, IQR [− 33; 9]. At month 3, 23 out of the 80 patients (28.8%) had a decrease in eGFR greater than 20 mL/min/1.73 m^2^. Overall, 23/80 patients (28.8%) had an estimated GFR below 60 mL/min/1.73 m^2^ at month 3 (CKD KDIGO stage 3 to 5), compared to 9/80 (11.2%) before hospital admission for rhabdomyolysis (*p* = 0.0009) (Fig. 2). One patient required dialysis at month 3. The severity of the AKI and serum myoglobin levels, higher than 8000 U/L at admission, were significantly associated with the change in CKD KDIGO stage at month 3 (*p* = 0.02 and *p* = 0.005, respectively).

## Discussion

Rhabdomyolysis is a frequent, life-threatening condition that develops solely or in combination with an underlying acute condition. Large studies specifically dedicated to severe rhabdomyolysis are scarce, and mainly rely on registries, thus precluding an accurate characterization of the predictive factors of severe AKI [5, 12, 18, 19].

In our series of 387 individuals from eight ICUs in France, immobilization, crush syndrome and vascular ischemia were the primary causes of severe rhabdomyolysis. In line with previous studies, it is worth noting that despite multiorgan failure, and the need of aggressive therapies as mechanical ventilation and vasoactive drugs, the mortality rate of the current cohort was relatively low (10.9% at day 28) [3, 18].

In recent years, epidemiological studies clearly demonstrated a significant risk of progression from AKI to CKD after a single event of AKI [20]. Contrasting with data from older studies with short follow-up times, we showed that CKD also develops after rhabdomyolysis-induced AKI. Approximately 30% of patients that survived the rhabdomyolysis had a significant decrease in eGFR at 3 months (i.e., greater than 20 mL/min/1.73 m^2^). Moreover, a significant proportion of patients developed overt CKD (i.e., eGFR below 60 mL/min/1.73 m^2^). This idea is in agreement with animal studies that have demonstrated that rhabdomyolysis-induced AKI was actually followed by progression to interstitial fibrosis and CKD [9]. Because the more severe patients were likely to have the longer follow-up, thus introducing a selection bias in our study, this needs to be confirmed in a prospective study to accurately address the risk of progression toward CKD after rhabdomyolysis.

In our series, the severities of the rhabdomyolysis and the AKI were two predictive factors of progression to CKD. In mice, macrophage depletion in the first few days can prevent the development of interstitial fibrosis. Beyond highlighting the need to develop immunomodulatory approaches to prevent or reverse rhabdomyolysis-induced AKI and CKD, our findings prompt to test how targeting the risk factors of severe AKI (for instance, serum phosphate) may improve the long-term renal outcome of patients that survive the severe rhabdomyolysis.

Risk factors of stage 2–3 AKI included the need for mechanical ventilation and the severity of the rhabdomyolysis at admission (i.e., serum phosphate, potassium and myoglobin levels at admission). As for other causes of massive cell lysis (i.e., tumor lysis syndrome), hyperphosphatemia was also accompanied by hypocalcemia and hyperuricemia. In our cohort, there was no correlation between serum phosphorus and creatinine at the admission suggesting that hyperphosphatemia was not only related to a decrease of the eGFR. The combination of these electrolyte disorders might contribute to the development of AKI [3, 5, 21]. Dramatic increase in phosphate levels, in the context of hypovolemia, might lead to acute deposition of calcium and phosphate within tissue, including the kidneys, with subsequent crystal-related acute tubular necrosis. Of note, serum phosphate levels at admission were not associated with changed eGFR at month 3. Also, we could not confirm the effectiveness of sodium bicarbonate to prevent severe AKI and the need of RRT. However, the retrospective design of our study deserves further interventional comparative studies.

Several recent studies reported that myoglobin may be efficiently removed from serum using intermittent hemodialysis or continuous veno-venous hemodiafiltration with high-cutoff membranes [22, 23]. Myoglobin promotes rhabdomyolysis-related AKI by reducing renal arterial blood flow, inducing proximal tubule toxicity, polarizing intrarenal pro-inflammatory macrophages and leading to intra-tubular obstruction. Interestingly, patients that received intermittent hemodialysis as a first modality of RRT had a better survival at day 28. Because of the retrospective design of our study, we could not address the relation between RRT modalities (membranes, timing, intermittent vs. continuous RRT) and the early removal of myoglobin or the subsequent AKI to CKD transition in patients at high risk of developing stage 2–3 AKI. This remains to be addressed in prospective interventional studies.

Notwithstanding the limitations of our study linked to its retrospective design, we reported here a large cohort with extensive characterization of the clinical and biological parameters at the admission to the ICU that may modify the renal outcomes. Because we focused on patient admitted to the ICU, heterogeneity of the admission policies may have biased the results by excluding oldest patients and patients with drugs-induced or autoimmune disease-related severe rhabdomyolysis without organ failure. However, our results obtained in a homogeneous population of patients admitted to the ICU will help to design a prospective interventional study in this setting.

## Conclusions

Severe rhabdomyolysis leads to AKI in most patients admitted to an ICU and requires RRT in a third of the cases. The long-term renal outcome appears to be strongly correlated to serum myoglobin and phosphate levels at admission, two molecules that might be removed using specific devices to reduce the risk of AKI to CKD progression.

## Data Availability

The datasets generated and analyzed during the current study are available from the corresponding author on reasonable request.
